# Etiology and Age Distribution of Syncope in a Predominantly African-American Population

**DOI:** 10.7759/cureus.1354

**Published:** 2017-06-14

**Authors:** Ahmad Awan, Hasan Iftikhar, Fasil Tiruneh, Shahabuddin Soherwardi, Daniel Larbi

**Affiliations:** 1 Department of Internal Medicine, Howard University Hospital

**Keywords:** syncope, african-american

## Abstract

**Background:**

Syncope, the sudden transient loss of consciousness due to multiple etiologies, usually has worse outcomes in African Americans compared with other races because of other comorbidities. This study aims to identify a correlation between the etiologies of syncope and age in a predominantly African-American sample population to facilitate future investigations with potentially improved specificity to address this condition.

**Methods:**

We reviewed the medical records of 155 patients who presented with syncope concerns to the emergency department at our university hospital. After three charts were discarded due to poor data, the patients were divided into four age groups: <40 years old (25 patients), 40–60 years old (62 patients), 61–80 years old (44 patients), and >80 years old (21 patients). The etiology of syncope was reviewed in each case and categorized as either a vasovagal episode, orthostatic hypotension, pulmonary embolism, and related to cardio, neurologic, drug, or unspecified.

**Results:**

For most of the patients in our study population, regardless of age, the etiology of syncope remained unspecified. Vasovagal related was the second most common etiology. The likelihood of having cardiac etiology under the age group of 40 is very low compared with those over 40 years (*p* = 0.026). Also, the incidence of pulmonary embolism is very low in all age groups with pulmonary embolism as a cause of syncope seen in only three patients (*n* = 3; 1.95%).

**Conclusions:**

Except for cardiac etiology being more likely in patients over age 40, we found no other correlation of age to syncope etiology. We also found that the etiology of syncope in African-American patients is similar to that of the general population. We recommend physicians to order a relevant workup, when clinical suspicion for syncope is present along with specific etiologies.

## Introduction

Syncope is defined as a sudden transient loss of consciousness, which could be due to multiple etiologies. The pathophysiology of all forms of syncope consists of a sudden decrease in or brief cessation of cerebral blood flow [1]. In the general population, there are 18.1–39.7 episodes of syncope per 1,000 patients, with a similar incidence between genders [2]. Syncope represents 3–5% of emergency department visits and 40% of cases lead to hospitalization. Although the exact cost of syncope is unknown, according to one conservative estimate, the annual cost for syncope-related hospitalization is $2.4 billion, with a mean cost of $5,400 per hospitalization [3].

The etiology can vary from benign to life-threatening. Compared with other races, African Americans have been reported to have the worst outcomes with syncope usually due to other comorbidities [Abstract: Pratap B, Bastawrose J, Pamidimukala C, et al.: African-American Patients Presenting with Unexplained Syncope Have Significantly Worse Outcome Compared to Other Races: An ACAP-SELF Dataset Analysis. Circulation: Cardiovascular Quality and Outcomes. 2014, 7:A376].

This study attempts to identify a correlation between the various etiologies of syncope and age in a predominantly African-American sample population to facilitate future investigations with potentially improved specificity to address this condition.

## Materials and methods

We performed a retrospective review of the medical records of 155 patients presenting to the emergency department at our university hospital with syncope concerns from January 2011 to December 2014. Three patients' medical records were excluded due to poor quality data. Data collected included the patient’s initial history, vitals, laboratory investigations, imaging studies, and discharge summaries. To be included in this study, patients must have had at least one episode of syncope, a typical history suggesting syncope, and be at least 15 years old. Patients were further characterized based on age, gender, ethnicity, and comorbidities. The final diagnoses were grouped into the following etiologies of syncope: cardiogenic, orthostatic hypotension, vasovagal, neurological, pulmonary embolism (PE), drug related, and undetermined etiology. Patients were classified into four different age groups. Group 1 contained patients aged 15–40 years, group 2 contained patients aged 41–60 years, group 3 contained patients aged 61–80 years, and group 4 patients were over 80 years old. Statistical analysis was performed using SAS® statistical analysis software (JMP®, Version 9. SAS Institute Inc., Cary, NC, 1989-2007) to determine the statistical significance based on etiology in different age groups.

## Results

Of the 152 patients in the study population, 128 patients (84.8%) were African-American (Table 1). Our sample size had a similar proportion of male and female patients (75 men and 77 women; Table 2). We identified comorbid conditions and presented them in Table 3.

**Table 1:** Racial distribution of study population (N = 152)

Group	Age Range (years)	African American (*n* = 128)	Caucasian (*n* = 14)	Asian (*n* = 5)	Hispanic (*n* = 5)
1	15–40	19	3	1	2
2	41–60	54	3	3	2
3	61–80	36	6	1	1
4	≥81	19	2	0	0

**Table 2:** Gender distribution of study population (N = 152)

Group	Age Range (years)	Male (*n* = 75)	Female (*n* = 77)
1 (*n* = 25)	15–40	12	13
2 (*n* = 62)	41–60	32	30
3 (*n* = 44)	61–80	23	21
4 (*n* = 21)	≥81	8	13

**Table 3:** Comorbid conditions in study population (N = 152) Abbreviations: DM, diabetes mellitus; Dx, disease; HTN, hypertension; Hx, history.

Group	Age Range	HTN	DM	Heart Dx	Psychiatric Hx	Seizure Hx	Pulmonary Dx
	(years)	(*n* = 92)	(*n* = 33)	(*n* = 49)	(*n* = 35)	(*n* = 10)	(*n* = 31)
1	15–40	2(8)	2(8)	5(20)	8(32)	2(8)	5(20)
2	41–60	37(60)	13(20)	18(29)	14(23)	3(4)	16(26)
3	61–80	34(77)	11(25)	17(38)	7(16)	5(11)	10(23)
4	≥81	19(90)	7(33)	9(43)	6(29)	0(0)	0(0)

Table 4 presents the etiology data categorized by age group. We had 25 patients in the 15–40 age group, and the etiology of syncope for this group was undetermined in nine patients (36%), vasovagal in eight patients (32%), orthostatic hypotension in six patients (24%), neurological in one patient (4%), and drug related in one patient (4%).

**Table 4:** Etiology of syncope by age group Abbreviations: PE, pulmonary embolism.

	Group 1	Group 2	Group 3	Group 4
	15–40 years	41–60 years	61–80 years	>81 years
Etiology	*n* (%)	*n* (%)	*n* (%)	*n* (%)
Idiopathic	9 (36)	25 (40)	16 (38)	10 (14)
Vasovagal	8 (32)	15 (24)	13 (30)	7 (33)
Orthostatic	6 (24)	8 (12)	3 (7)	1 (4)
Cardiac	0 (0)	6 (14)	4 (9)	3 (14)
Neurology	1 (4)	1 (1)	4 (9)	0 (0)
Drug Related	1 (4)	5 (8)	2 (5)	0 (0)
PE	0 (0)	1 (1)	2 (5)	0 (0)

Sixty-two patients were in the 41–60 years group. The etiology of syncope for this age group was undetermined in 25 patients (40%), vasovagal in 15 patients (24%), orthostatic hypotension in eight patients (12%), cardiogenic in six patients (9%), drug related in five patients (8%), neurological in one patient (1%), and PE in one patient (1%).

Forty-four patients were in the 61–80 years group, and syncopal etiology was undetermined in 16 of them (38%). Etiologies were vasovagal in 13 patients (30%), cardiogenic in four patients (9%), neurological in four patients (9%), orthostatic hypotension in three patients (7%), PE related in two patients (5%), and drug related in two patients (5%).

Twenty-one patients were in the >80 years group. Once again, the etiology of syncope was undetermined in 10 patients (47%) in this group, vasovagal in seven patients (33%), cardiogenic in three patients (14%), and orthostatic hypotension related in one patient (4%).

The incidence of cardiac-related etiology for syncope in patients under age 40 was statistically significantly lower than those patients over age 40 (*p* = 0.026). However, no statistically significant difference was noted between age groups for vasovagal (*p* = 0.093), orthostatic hypotension (*p* = 0.2319), neurological causes (*p* = 0.087), PE (*p* = 0.2056), and drug related (*p* = 0.1746).

## Discussion

The evaluation and management of patients with syncope is one of the more challenging issues in clinical practice [4]. According to the literature, African Americans are more likely to have multiple comorbidities, which translate to poorer outcomes of syncope [3]. Although many underlying causes of syncope are benign, others are associated with substantial morbidity or mortality, including cardiac arrhythmia, myocardial infarction, PE, and occult hemorrhage.

Syncope is not a disease; rather, it is a symptom, which could be present in a variety of diseases. To the best of our knowledge, no study has evaluated the correlation of the etiology of syncope with age.

In most studies, no etiology is found [1]. Even in our study, despite routine investigations, the number one etiology in all age groups was idiopathic. In these cases, patients should be managed symptomatically. While a thorough history and physical examination are certainly warranted when the etiology is idiopathic, relevant investigations should be ordered only if warranted based on the patient’s history and physical examination.

In cases where the etiology of syncope is not idiopathic, vasovagal syncope was the most commonly identified cause [5]. The mechanism of vasovagal syncope is explained by the Bezold-Jarisch reflex, which is triggered by decreased venous return, resulting in inadequate ventricular filling and vigorous cardiac contraction [2]. Vasovagal syncope usually shows bimodal distribution and is the most common cause of syncope in young people. Our findings align with the literature; the prevalence of vasovagal syncope ranged from 24% to 32% in our study. The difference between age groups was not statistically significant (*p* = 0.093).

The diagnosis of syncope is usually based on patient history and physical examination. Therapy is directed at identifying and treating the underlying precipitating factor. Non-pharmacological measures include having the patient assume the supine position during the time of prodromes and supplementing the patient’s diet with nutritional supplements and sodium [2]. An extensive workup is not recommended in patients presenting with their first episode of vasovagal syncope. However, in recurrent cases or in patients in which the etiology is doubtful, tilt table testing is indicated. Tilt table testing helps differentiate vasovagal syncope, orthostatic hypotension, and postural tachycardia syndrome.

A common concern in syncope patients, especially those with structural heart disease, is arrhythmia. Testing for arrhythmias includes recording electrocardiographic data using a Holter monitor or other electrophysiologic studies, which are not only expensive, but also cumbersome for the patient, requiring prolonged hospital stays or multiple clinic visits. Our research shows that the likelihood of cardiac etiology in people under 40 years old is low, and the likelihood is statistically significantly greater (*p* = 0.026) in patients over age 40. This could be secondary to a low incidence of hypertension and heart disease in the younger age group. With our findings, we recommend that extensive workups may be avoided in patients younger than 40 unless the patient has strong risk predictors for heart disease.

Orthostatic hypotension: low blood pressure resulting from moving to a standing position from a sitting or lying position. It occurs when a patient’s systolic blood pressure falls 20 mm of Hg or his diastolic blood pressure falls 10 mm within two minutes of quiet standing [6]. According to the literature, the incidence of orthostatic hypotension gradually increases with age and is more prevalent in the elderly population due to impaired baroreceptor sensitivity [7-8]. Epidemiologic surveys have found postural hypotension in up to 20% of patients over age 65 [9-10].

Our findings indicate that orthostatic hypotension is an important cause of syncope in all age groups, with a prevalence ranging from 4% to 24%. However, the difference between the groups was not statistically significant (*p* = 0.2319).

In nearly 10% of cases reported in the literature, the cause of syncope is a neurological disorder [1]. However, the age-wise distribution of neurological causes has not been extensively studied. We found no statistically significant difference between age groups for neurological causes of syncope (*p* = 0.087). When neurological causes of syncope are suspected, physicians should consider further workups. However, multiple studies have shown that routine use of computed tomography (CT) imaging of the head is not useful in elucidating the etiology of syncope.

PE is an important differential for syncope. The incidence of PE as the cause of syncope is unknown. However, a recent study reported that incidence can be as high as 17.3% [11]. For patients with a low Wells score, a D-dimer is ordered. However, a diagnosis can only be confirmed with either CT angiography or a ventilation-perfusion scan, both of which are expensive tests. In our study, the overall incidence of PE is less than 3% with just three cases noted in patients aged 41–80 years. The treatment for PE is usually anticoagulation therapy, and thrombolytic treatment is recommended in unstable patients.

Our study had several limitations. We had a small sample size, given that our population was culled from a single suburban hospital in Washington, DC. The focus on African-American patients also means that our findings may not generally be applicable to other population subgroups.

## Conclusions

Except for cardiac etiology being more likely in patients over age 40, we found no other correlation of age to syncope etiology. Overall, we found that the etiology of syncope in African-American patients is similar to that of the general population. Our analysis indicates that a workup may not determine the etiology of syncope in most of the cases. We recommend physicians to order a relevant workup, when clinical suspicion for syncope is present along with specific etiologies. However, workups to rule out pulmonary embolism may be too restrictive of the full clinical picture with our findings of low pretest probabilities for pulmonary embolism in our syncope patient population.
